# ERK Phosphorylation Regulates Sleep and Plasticity in *Drosophila*

**DOI:** 10.1371/journal.pone.0081554

**Published:** 2013-11-14

**Authors:** William M. Vanderheyden, Jason R. Gerstner, Anne Tanenhaus, Jerry C. Yin, Paul J. Shaw

**Affiliations:** 1 Department of Anesthesiology, University of Michigan Medical School, Ann Arbor, Michigan, United States of America; 2 Department of Anatomy, Washington University Medical School, Saint Louis, Missouri, United States of America; 3 Translational Research Laboratories, Center for Sleep and Circadian Neurobiology, Perelman School of Medicine, University of Pennsylvania, Philadelphia, Pennsylvania, United States of America; 4 Departments of Genetics and Neurology, University of Wisconsin, Madison, Madison, Wisconsin, United States of America; Rutgers University, United States of America

## Abstract

Given the relationship between sleep and plasticity, we examined the role of Extracellular signal-regulated kinase (ERK) in regulating baseline sleep, and modulating the response to waking experience. Both sleep deprivation and social enrichment increase ERK phosphorylation in wild-type flies. The effects of both sleep deprivation and social enrichment on structural plasticity in the LNvs can be recapitulated by expressing an active version of ERK (*UAS-ERK* ^*SEM*^) pan-neuronally in the adult fly using GeneSwitch (*Gsw*) *Gsw-elav-GAL4*. Conversely, disrupting ERK reduces sleep and prevents both the behavioral and structural plasticity normally induced by social enrichment. Finally, using transgenic flies carrying a cAMP response Element (CRE)-luciferase reporter we show that activating ERK enhances CRE-Luc activity while disrupting ERK reduces it. These data suggest that ERK phosphorylation is an important mediator in transducing waking experience into sleep.

## Introduction

Waking experience, including both prolonged wakefulness and exposure to enriched environments, independently produce dramatic increases in both synaptic markers and structural morphology throughout the fly brain and these changes are reversed during sleep [1-3]. A strategy that has been useful for identifying the molecular mechanisms by which waking experience can increase sleep has been to evaluate mutations that disrupt synaptic plasticity [2,4]. For example, flies mutant for the adenylyl cyclase *rutabaga* (*rut* ^*2080*^), the clock gene period (per), the *Drosophila* homologue of *Serum Response Factor*, *blistered* (*bs*), and Epidermal growth factor receptor (Egfr), have deficits in plasticity and do not respond to enriched environments with increases in sleep [4]. Importantly, rescue of each of these genes within the Pigment Dispersing Factor (PDF)-expressing ventral lateral neurons (LNvs) restores both plasticity and increased sleep following social enrichment. Similarly, over-expression of the *fragile X mental retardation 1* (*Fmr1*) gene, which promotes neural pruning [5], blocks increases in the number of dendritic spines [1]. Interestingly, while genetic manipulations that disrupt the formation of synapses have been evaluated for their role in sleep regulation, it is currently unknown how genetic manipulations that drive synaptic plasticity influence sleep.

Extracellular signal-regulated kinase (ERK) is a key molecule for the regulation of synaptic plasticity and is conserved throughout phylogeny [6-12]. In rodents, ERK phosphorylation is necessary for learning [13], including conditioned place preference [14], fear conditioning [15] and spatial learning [16]. The *Drosophila*, homologue of ERK, *rolled* (*rl*), has been shown to regulate synaptic bouton number at the larval neural muscular junction [17,18] and has recently been correlated with sleep time following *rhomboid* and Star mediated activation of the EGFR [19]. Moreover, ERK has been shown to regulate cAMP response element binding protein (CREB) [20], a transcription factor that couples synaptic activity to long-term changes in neuronal plasticity [21] .

We show sleep deprivation and social enrichment independently increase ERK phosphorylation in wild-type flies. We also report that expressing an active version of ERK (*UAS-ERK* ^*SEM*^) in the adult fly results in an increase in sleep and an increase in structural plasticity in the LNvs. Conversely, disrupting ERK reduces sleep and prevents both the behavioral and structural plasticity normally induced by social enrichment. Finally, using transgenic flies carrying a cAMP response element (CRE)-luciferase reporter (CRE-Luc) we show that ERK activation enhances CRE-Luc activity while disrupting ERK reduces it. These data suggest that phosphorylation of ERK may be a mechanism for regulating sleep and plasticity. 

## Methods

### Fly husbandry and sleep deprivation

Flies were cultured at 25°C, 50-60% humidity, maintained on a 12:12 Light:Dark schedule, on yeast, sucrose, molasses, corn syrup and agar food. Newly eclosed adult flies were collected from culture vials daily under CO_2_ anesthesia. Three day old flies were placed into 65mm glass tubes and sleep parameters were continuously evaluated throughout the remainder of the experiment using the Trikinetics activity monitoring system as described previously [22,23]. Female flies were used for all experiments. Flies were sleep deprived using an automated sleep deprivation apparatus that has been found to produce waking without activating stress responses. The Sleep Nullifying Apparatus (SNAP) tilts asymmetrically from -60° to +60° such that sleeping flies are displaced during the downward movement 6 times per minute while being monitored in the Trikinetics monitor. This stimulus is effective presumably because it initiates a geotactic response [22,23]. Sleep homeostasis was calculated for each individual as a ratio of the minutes of sleep gained above baseline during recovery divided by the total min of sleep lost during sleep deprivation (min gained/min lost).

### Fly stocks

*UAS-ERK* ^*SEM*^ flies, as published in [24], were obtained as a generous gift from the lab of Dr. Aaron Diantonio (Washington University in Saint Louis). *GSw-elav, rut* ^2080^ *, rl* ^1^ and *UAS-RSK* ^*wt*^ were obtained from the Bloomington Stock Center (Bloomington, Indiana). Wild type Canton s (Cs) flies are flies maintained in house as the lab wild-type strain. Flies used for the CRE-Luc experiments are described in [25].

### Social enrichment

To standardize the environmental conditions during critical periods of brain development, virgin female flies were collected upon eclosion and maintained in same-sex vials containing 30 flies for 3 days. This protocol keeps environmental conditions constant between subsequently isolated and enriched flies for the first 3 days of adult life. Three to four day old flies were then divided into a socially isolated group, which were individually housed in 65-mm glass tubes, and a socially enriched group, consisting of 45-50 female flies housed in a single vial as previously described [4]. After five days of social enrichment/isolation, flies were placed into clean 65-mm glass tubes and sleep was recorded for three days using the Trikinetics activity monitoring system. To calculate the mean and standard error for ∆Sleep in the experimental group we first calculate the mean of the daytime sleep for the isolated group, averaged over three days, and then subtracted it from the average daytime sleep observed for each individual socially-enriched sibling. The difference is referred to as ∆Sleep.

### Immunohistochemistry

For the sleep deprivation and social enrichment studies, all animals were sacrificed at ZT0 and immediately processed for either immunohistochemistry or western blotting. Brains were dissected in cold PBS and processed for standard whole-mount immunostaining as described previously [26,27]. The following antibodies were used: Guinea Pig anti-PDF (Gift from Dr. Paul Taghert; Washington University in Saint Louis) at 1:1000, mouse anti-ppERK (Sigma) at 1:1000, and Alexa 488 conjugated anti-mouse and anti-Guinea Pig IgG (Molecular Probes) at 1:1000. Confocal images were collected on an Olympus microscope provided by the Bakewell Neuro-imaging Facility at Washington University in Saint Louis. Confocal stacks of PDF terminals were quantified as described previously [4]. Briefly, immuno-positive terminals were counted using the ImageJ binary thresholding algorithm. The number of synaptic terminals for the socially isolated flies was used to generate a mean. The mean of the isolated flies was used to normalize each individual enriched brain. The individual normalized values were then used to calculate the mean and standard error for the group. The mean and standard error for socially isolated flies were calculated by normalizing to their own group mean. The normalized values for each group were then evaluated using an independent sample t-test.

### Western blot

Four fly heads per group were homogenized in 15µl Sample Buffer (4% SDS, 20% glycerol, 10% 2-mercaptoethanol, 0.004% bromphenol blue and 0.125 M Tris HCl, pH approx. 6.8 (Sigma Aldrich, St Louis, MO.)) then heated to 100 degrees Celsius for 5 minutes and then centrifuged at max speed for 3 minutes and loaded (12.5 ul) on the gel (10% TGX (Biorad)). Gel was run at 200v for 30 minutes. Gel was transferred to PVDF membrane at 4 degrees Celsius on ice at 100v for 1.5 hours. Blot was probed with mouse anti-ppERK antibodies (Sigma Aldrich, St Louis, MO.) 1:1000, rabbit anti-total ERK antibodies (Sigma Aldrich, St Louis, MO.) 1:1000. Blot was visualized using a Biorad chemiluminescence detector and quantified using ImageJ software (NIH). For the nuclear localization studies, 40 heads were homogenized in extraction buffer (15mM HEPES pH 7.5, 10mM KCl, 5mM MgCl2, 0.1mM EDTA, 0.5mM EGTA, plus protease inhibitors and DTT) and spun on a centrifuge so as to separate the cytosolic and nuclear fractions using techniques published previously [28].

### Pharmacology

The drug SL327 (Sigma Aldrich, St Louis, MO.), a MAP Kinase Kinase (MEK) inhibitor, was added to melted food at a 2mM concentration, vortexed briskly in order to form an emulsion, and allowed to cool to room temperature. Similarly, RU486 (Sigma Aldrich, St Louis, MO.) was dissolved in 100% Ethanol at a concentration of 50mg/mL and added to melted fly food for a final concentration of 100µg/mL as previously published [26].

### CRE-Luc

CRE-Luc cycling activity was assayed as previously described [25]. 3-day-old *CRE-Luc* flies were placed individually into single wells of a 96-well plate containing 100:l of a 5% sucrose−agar mixture, with added luciferin to a final concentration of 5 mM. Flies were then placed in the Topcount (Packard, Meridan, Connecticut) luminometer in LD at 25°C. Bioluminescence was measured hourly in each plate (18 s/well or 36 s/well averaged to obtain a final count), for three consecutive days. 

## Results

### Baseline sleep time is regulated by ERK activation

**Figure 1:**
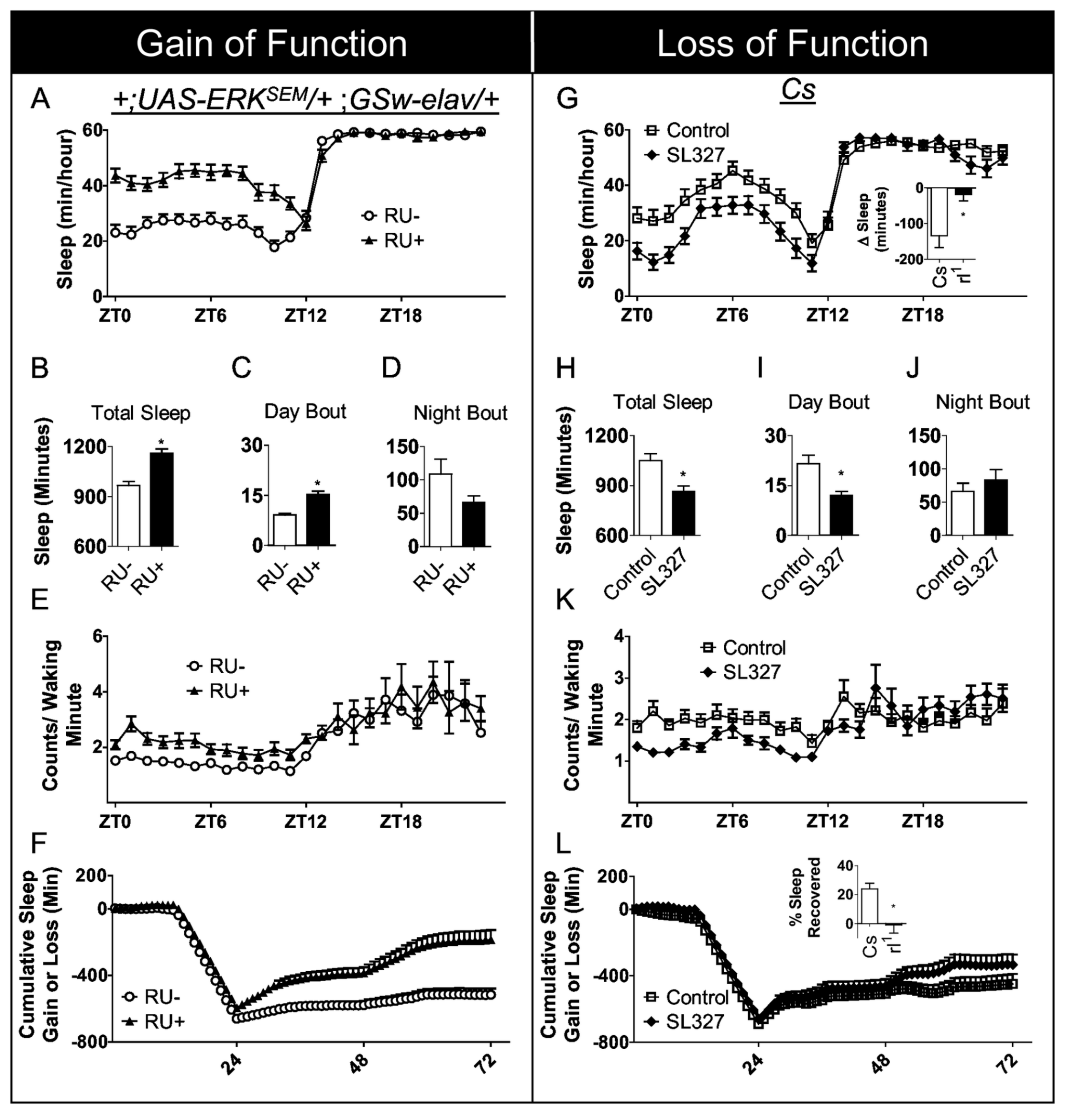
ERK activation increases sleep. *A*, Pan-neuronal expression of activated ERK in adult flies results in an increase in sleep. Sleep is shown in minutes/hour in RU486 (RU+) fed *+*;*UAS-ERK^SEM^/+* ;*GSw-elav/+* flies and their vehicle fed controls (RU-). A 2(RU+, RU-) X 24(Hour) ANOVA reveals a significant drug by hour interaction F_(23,1488)_ = 7.42, *P* = 5.1E-23,(n = 32/ group). *B*-*D*, Total sleep time and sleep bout duration during the light period were significantly increased in RU+ fed *+*;*UAS-ERK^SEM^/+* ;*GSw-elav/+* compared to RU- controls, p = 7.25E-7 and p = 1.77E-6, respectively; student’s t-test, n=31-32/group. RU+ treated flies showed no change in sleep consolidation at night p = 0.103; student’s t-test, n=31-32/group. Data are presented as mean ± SEM, (* = p < 0.05). *E*, RU fed *+*;*UAS-ERK^SEM^/+* ;*GSw-elav/+* showed increased activity during waking (ZT1-ZT12) compared to vehicle fed controls; A 2(RU+, RU-) X 12(Hour) ANOVA reveals a significant main effect of drug *F*_(1,600)_ = 88.47, *P* = 1.08 E-19] (n = 26/ group). *F*, RU+ fed *+*;*UAS-ERK^SEM^/+* ;*GSw-elav/+* showed an exaggerated homeostatic response following 12 h of sleep deprivation compared to RU- fed siblings. Data are presented as cumulative loss then gained in minutes. A negative slope indicates sleep lost, and a positive slope indicates sleep gained; when the slope is zero, recovery is complete. A 2(RU+, RU-) X 72(Hour) ANOVA reveals a significant drug by hour interaction [F_(71,3888)_ = 8.08, *P* = 2.88E-73] (n = 28/ group). *G*, Pharmacological inhibition of ERK in adult *Cs* flies reduces sleep. Adult flies were fed 2mM of the Mitogen-activated protein kinase kinase (MEK) inhibitor SL327. Two-way ANOVA reveals a significant interaction of drug (SL3227, control) treatment by time [*F*(_23,1392_) = 9.57, *P* = 2.4E-31] (n = 30 for each group). Inset shows that SL327 administration reduces total sleep time significantly in wild type *Cs* flies but did not alter total sleep in the *Drosophila rl* ^1^ mutants p= 0.08 (shown in inset), suggesting specificity of the drug to cause the sleep reduction phenotype. *H*-*J*, Total sleep time and sleep consolidation during the light period were significantly decreased in SL327 fed flies (n=30) compared to vehicle fed controls (n=30), p = 0.0008 and p = 0.0014, respectively; student’s t-test. SL327 did not alter sleep consolidation at night, p = 0.379, (* = p < 0.05). *K*, Activity during waking (ZT1-ZT12) is significantly reduced in adult *Cs* flies fed SL327 compared to vehicle fed controls; ; A 2(RU+, RU-) X 12(Hour) ANOVA reveals a significant main effect of drug [*F*_(1,744)_ = 57.74, *P* = 9.01E-14 (n = 32 flies)]. *L*, No change in sleep homeostasis was observed in SL327 fed *Cs* flies compared to vehicle fed controls; 2(SL327, control) X 72(Hour) ANOVA,[F_(71,3024)_ = 0.393, *P* = 0.999 (n = 22/ group)].

To determine whether increased ERK activity plays a causative role in regulating sleep, we expressed a constitutively active form of ERK (*UAS-ERK* ^*SEM*^) pan-neuronally in adult flies using GeneSwitch *elav GAL4* (*GSw-elav*) [29]. As seen in Figure 1A, RU (RU+) treated *+*;*UAS-ERK* ^*SEM*^ */+* ;*GSw-elav/+* flies displayed a significant increase in sleep compared to vehicle (RU-) fed siblings. On average, flies expressing *UAS-ERK* ^*SEM*^ pan-neuronally exhibited ~2-3 h more sleep than untreated controls (Figure 1B). The increase in sleep was associated with an increase in sleep bout duration during the day (Figure 1C), but not during the night (Figure 1D). This phenotype was not dependent on light or circadian input as well. RU+ treated *+*;*UAS-ERK* ^*SEM*^ */+* ;*GSw-elav/+* flies show a similar increase in sleep compared to RU- fed controls in Dark:Dark conditions (Figure S1A). Total sleep was not altered in RU+ fed *Gsw-elav/+* or *UAS-ERK* ^*SEM*^ */+* background parental lines compared to vehicle controls (RU-) (n =28-30/group, t-test, p=0.23 and p=0.28, respectively, Figure S2). To determine whether expressing *UAS-ERK* ^*SEM*^ resulted in a weak or sick fly, we examined the intensity of waking activity. As seen in Figure 1E, RU-fed *+*;*UAS-ERK* ^*SEM*^ */+* ;*GSw- elav/+* flies displayed a significant increase in waking activity indicating that they retained intact locomotor functioning (Figure 1E). Importantly, sleep episodes were rapidly reversible indicating that ERK activation did not simply induce a comatose-like state (Arousal thresholds shown in Figure S3).To determine whether flies would defend their increased sleep, we assessed homeostatic regulation following 12 h of sleep loss. As seen in Figure 1F RU+ treated *+*;*UAS-ERK* ^*SEM*^ */+* ;*GSw-elav/+* flies exhibited a significantly larger sleep rebound while vehicle treated (RU-) controls displayed a homeostatic response similar to that typically seen in wild-type flies [22]. To determine whether ERK activation in previously identified sleep/wake regulating circuits would modify sleep time, we expressed *UAS-ERK* ^*SEM*^ in the LNvs, Mushroom Bodies, the dorsal Fan Shaped body, and the pars intercerebralis [4,19,30-34]. As seen in Table 1, ERK activation did not alter sleep when expressed with these *GAL4* drivers compared to both parental controls. Together, these data suggest that ERK may be working in an as yet unidentified sleep regulatory circuit, may be required in multiple circuits to alter sleep, or that ERK is working in a non-cell autonomous fashion by modifying global brain activity. Nonetheless, these data show that constitutive pan-neuronal activation of ERK in the adult is sufficient to increase total sleep time. 

**Table 1:** ERK over-expression in previously determined sleep/wake circuits in *Drosophila*.

	GENOTYPE	TST (mean±sem)
	UAS-ERKact / +	763±13
MB	c309 / +	812 ± 15
	c309 / uas ERKact	795 ± 23
	201y / +	621 ± 28
	201y / uas ERKact	519 ± 22
	p247 / +	717 ± 33
	p247 / uas ERKact	657 ± 24
	ok107 / +	770 ± 34
	ok107 / uas ERKact	739 ± 26
PI	c767 / +	758 ± 19
	c767 / uas ERKact	720 ± 17
	c687 / +	767 ± 21
	c687 / uas ERKact	676 ± 18
	50y / +	782 ± 24
	50y / uas ERKact	700 ± 20
Clock	PDF GAL4 / +	908 ± 31
	PDF GAL4 / uas ERKact	733 ± 22
	c929 / +	815 ± 24
	c929 / uas ERKact	676 ± 19
	Tim Gal4 / +	660 ± 25
	Tim Gal4 / uasERKact	748 ± 18
CC	104y / +	713 ± 30
	104y / uas ERKact	801 ± 17
	c5 / +	637 ± 37
	c5 / uas ERKact	668 ± 30

Table shows baseline total sleep time in flies that are over expressing *UAS-ERK* ^*SEM*^ in the following previously determined sleep/wake circuits in the fly brain: Mushroom Bodies (MB), Pars Intercerebralis (PI), clock cells (Clock) or Central Complex (CC). Means and SEM are listed for the over-expressing flies and their parental lines with no statistically significant change due to *UAS-ERK* ^*SEM*^ over-expression with the exception of the 201y driven *UAS-ERK* ^*SEM*^ (*F(2,86*)*=31.6, P* = 5.8E-11 (n=26-32/group) One way ANOVA).

Next, we asked whether disrupting ERK activation in the adult would reduce sleep. Wild-type Canton s (Cs) flies were fed the MAP Kinase Kinase (MEK) inhibitor, SL327. MEK is a dual-specificity kinase that specifically activates ERK such that when MEK is inhibited, ERK phosphorylation is disrupted [35]. We chose a pharmacological approach to alter ERK in adults because, in our hands, the available *UAS-ERK-* ^*RNAi*^ lines did not reduce ERK transcript levels using any *GAL4* drivers, including *Gsw-elav GAL4* (data not shown). As seen in Figure 1 G-I, flies fed SL327 showed significantly reduced total sleep and shortened sleep episodes during the day. Administration of SL327 at this concentration was found to elicit statistically significant reductions in sleep. Lower doses of the drug did not reduce sleep to a significant level (Figure S4) and larger doses did not further reduce sleep. Importantly, SL327 did not reduce sleep in hypomorphic mutants for *Drosophila* ERK (*rolled*, *rl* ^1^) (Figure 1G, inset). Thus, the reduction in sleep observed in SL327-fed *Cs* flies is not likely a consequence of off-target effects of the drug. Similar to that observed with ERK overexpression, disrupting ERK activation with SL327 did not alter sleep bout duration at night (Figure 1J). Interestingly, while ERK activation increased waking activity, reducing ERK activity decreased locomotion during waking (Figure 1K). To test whether pharmacological inhibition of ERK would attenuate sleep homeostasis, *Cs* flies were maintained on SL327, sleep deprived for 12 h, and % sleep recovered was calculated during 48 h of recovery. Although SL327 did reduce sleep time during baseline, it was not able to lower the sleep rebound (Figure 1L). Given that ERK activation results in a larger sleep rebound, we proceeded to evaluate sleep homeostasis in the hypomorphic *rl* ^1^ mutants. The *rl* ^1^ mutant was first described in 1925 [36] and is a well characterized mutation that functions as a hypomorphic allele during development [37,38]; *rl* ^1^ flies sleep within the range observed in wild type flies (1034±28min) [39]. As seen in the inset in Figure 1L, *rl* ^1^ mutants did not exhibit a sleep rebound. The difference in the response to sleep deprivation that is observed between SL327-fed flies and *rl* ^1^ mutants could be due to developmental modifications in *rl* ^1^ mutants or incomplete pharmacological inhibition of ERK-activity following sleep deprivation. In any event, together these data suggest that ERK activation increases both daytime sleep and sleep consolidation while pharmacological inhibition of ERK reduces daytime sleep and shortens sleep bout duration during the day. 

### Sleep deprivation increases ERK activation and synaptic plasticity

**Figure 2:**
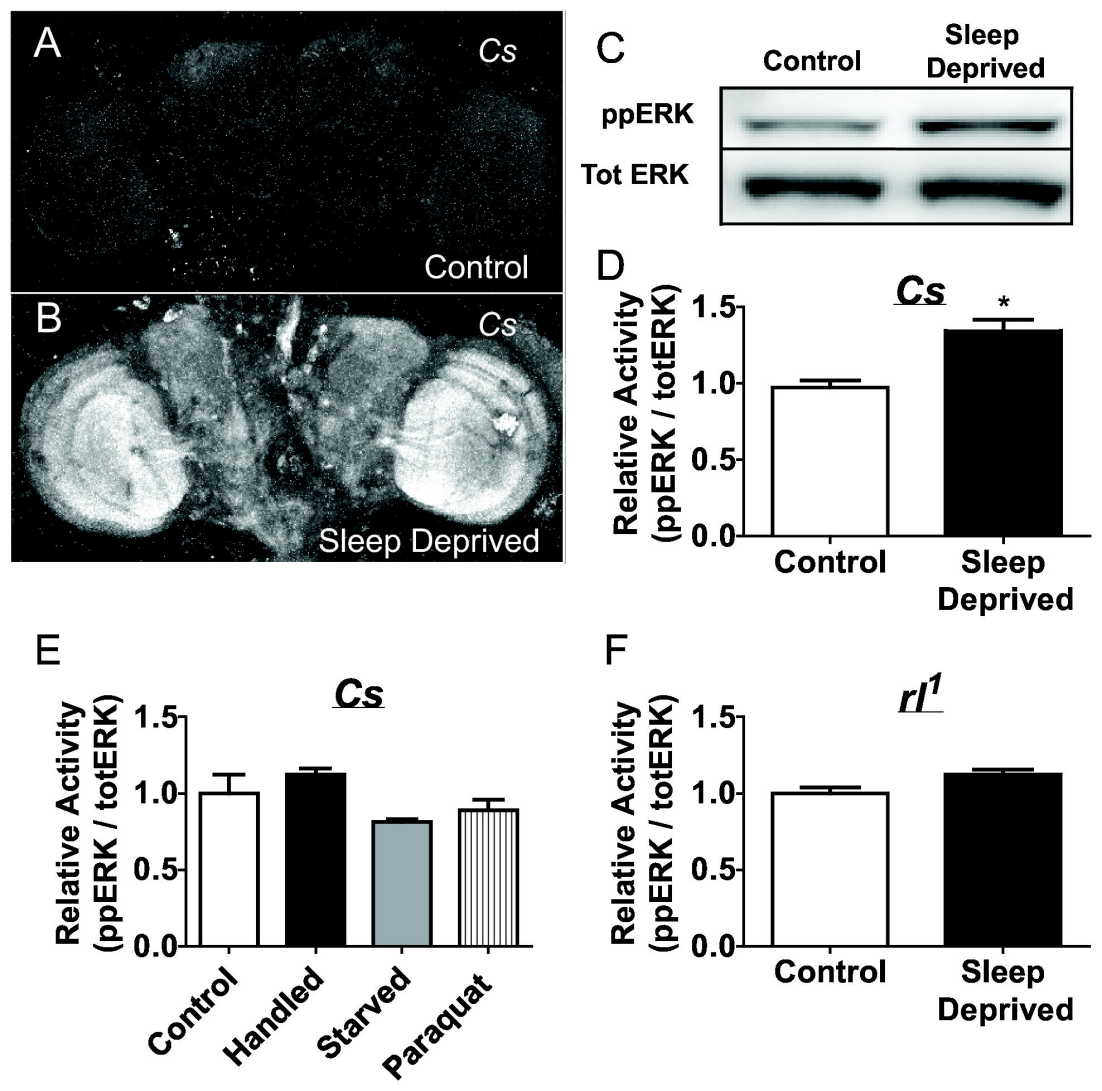
Sleep deprivation activates ERK. *A*-*B*, Immunohistochemistry of ppERK in brains from 5 day old *Cs* flies that experienced either a full night of sleep (*A*) or 12 hours of sleep deprivation (*B*). *C*, Western blot showing an increase in ppERK activation following 12 h of sleep deprivation in 5 day old *Cs* flies (4 heads/lane). *D*, Quantification of the Western blot in ***C*** revealed a statistically significant increase in ppERK/TotERK levels , p = 0.0059, student’s t-test, n= 4/group. Data are presented as mean ± SEM. *E*, Quantification of ppERK levels in wild type *Cs* flies exposed to mechanical perturbation (handled), the oxidative stressor Paraquat or starvation show no statistically significant differences in ppERK/total ERK levels compared to non-manipulated control flies p = 0.402, p = 0.477 and p = 0.209 respectively, student’s t-test, n = 3/group. Data are presented as mean ± SEM (* = p < 0.05). *F*, Quantification of the change in ppERK levels using Western blot in *rl* ^1^ mutant flies following 12 hours of sleep deprivation revealed no alteration in ppERK levels p = 0.0815, student’s t-test, n = 3/group. Data are presented as mean ± SEM.

To more fully evaluate the role of ERK in response to forced waking, we examined phosphorylated ERK (ppERK) levels after 12 hours of sleep deprivation. Wild-type *Cs* flies were sleep deprived for 12 h during their primary sleep period; their brains were removed and then assayed for phosphorylated ERK levels using whole mount immunohistochemistry. As seen in Figure 2A-B sleep deprivation dramatically increased levels of ppERK throughout the fly brain compared to non-sleep deprived circadian matched controls. We quantified changes in ppERK following sleep deprivation using Western blot analysis. As seen in Figure 2C-D, sleep deprivation resulted in a significant increase in ppERK levels relative to total ERK (totERK). Importantly, no changes in ppERK were observed when flies were exposed to 1) mechanical perturbation during the day, a manipulation that was designed to expose animals to the deprivation-stimulus without altering sleep homeostasis [23,40,41], 2) exposure to the toxin paraquat, or 3) starvation, a manipulation that is known to induce periods of waking without producing a sleep rebound or cognitive impairments [42,43] (Figure 2E). Consistent with these results, sleep deprivation did not modify ppERK levels in hypomorphic *rl* ^1^ mutant flies (Figure 2F). Additionaly, *Cs* flies maintained in Dark:Dark and deprived of sleep for 12 hours show the same increase in ppERK levels as those on a 12:12 Light:Dark schedule (Figure S1B,C). Together these data indicate that the increases in ppERK levels seen following sleep loss are not due to non-specific effects of stress or circadian effects. 

**Figure 3:**
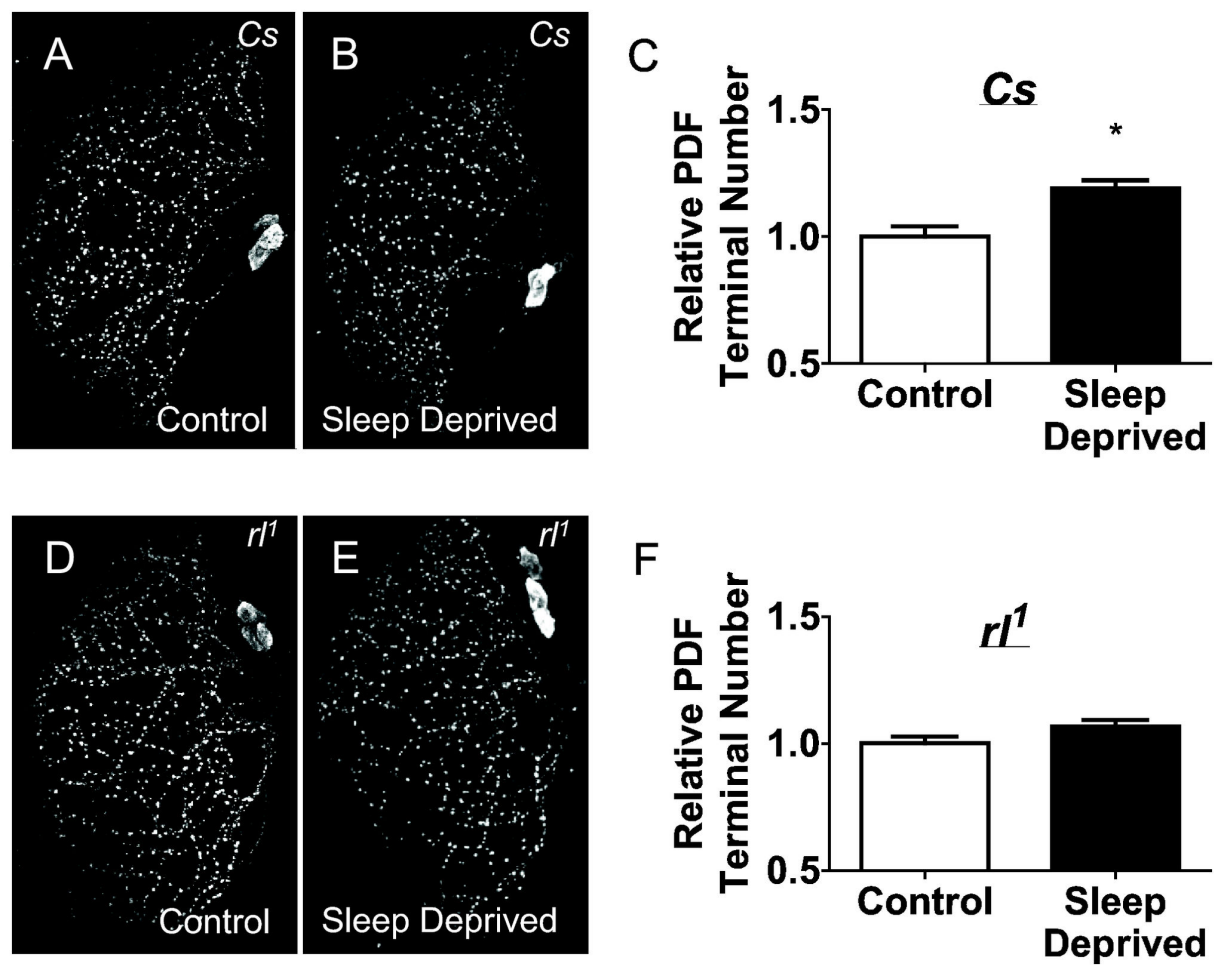
Sleep deprivation alters structural plasticity. *A*-*B*, Immunohistochemistry of pigment dispersing factor (PDF) in brains of wild-type *Cs* flies that experienced either a full night of sleep (*A*) or 12 hours of sleep deprivation (*B*). *C*, Quantification of PDF immunohistochemistry in *A*-*B*. 12 h of Sleep deprivation significantly increases PDF-positive terminals, p = 0.0011, student’s t-test, n= 13-14/group. Values normalized to non-sleep deprived controls and presented as mean ± SEM (* = p < 0.05). *D*-*E*, Immunohistochemistry of pigment dispersing factor (PDF) in brains of *rl* ^1^ mutant flies that experienced either a full night of sleep (*D*) or 12 hours of sleep deprivation (*E*). *F*, 12 h of sleep deprivation did not alter the number of PDF-positive terminals, p = 0.083, student’s t-test, n= 16-17/group. Values normalized to non-sleep deprived controls and presented as mean ± SEM (* = p < 0.05).

Previous studies have shown that sleep deprivation strongly up-regulates synaptic markers and is a potent modulator of synaptic morphology [1,3,4]. Given the role of ERK in neuronal plasticity we asked whether the increase in ppERK following sleep deprivation would modulate the number of pigment dispersing factor (PDF) positive terminals. As seen in Figure 3A-C, 12 h of sleep deprivation significantly increased the number of PDF-positive terminals in *Cs* flies compared to circadian matched non-sleep deprived controls. In contrast, sleep deprivation did not increase the number of PDF-positive terminals in *rl* ^1^ mutants (Figure 3D-F). These data suggest that sleep deprivation induced changes in ppERK may be responsible, in part, for changes in synaptic morphology.

### Social enrichment increases ppERK levels

**Figure 4:**
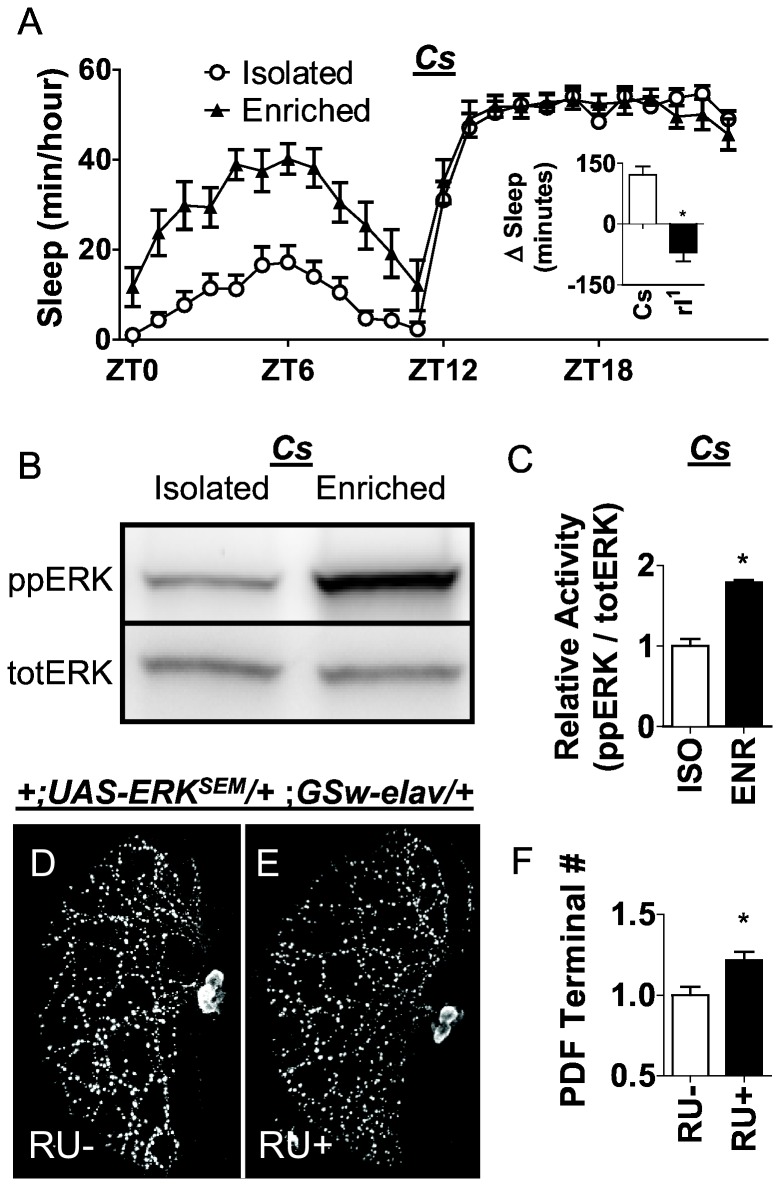
ERK increases structural plasticity. *A*, *Cs* flies exposed to 5 days of social enrichment with ~45 siblings display an increase in sleep compared to isolated controls. A 2(Isolated, Enriched) X 24 (Time (hour)) ANOVA reveals a significant condition x time interaction [*F*_(23,720)_ = 5.044, *P* = 3.6E-13] (n = 16/ group). The inset shows that this increase in sleep following social enrichment is attenuated in *rl* ^1^ mutant flies (shown as the change in sleep from the isolated siblings also known as ∆sleep). *B*, Representative Western blot showing that ppERK/totERK levels are increased in *Cs* flies following social enrichment compared to isolated siblings (4 heads per lane). *C*) Quantification of the Western blot in ***B*** for ppERK/totERK reveal a statistically significant increase in ppERK/totERK levels in the socially enriched group compared to isolated siblings p = 0.0002, student’s t-test. Data are presented as mean ± SEM (* = p < 0.05). *D*-*E*, Pan-neuronal expression of activated ERK in adult flies results in an increase number of PDF-positive terminals as revealed by immunohistochemistry. A representative image is shown for RU- fed *+*;*UAS-ERK^SEM^/+* ;*GSw-elav/+* controls (*D*) and their RU+ fed experimental group (*E*). *F*, Quantification of terminal numbers reveals a significant increase in terminal number p = 0.0076, n= 11/group, student’s t-test. Values normalized vehicle fed controls.

Recent studies have shown that exposure to enriched environments can increase daytime sleep and synaptic morphology in the absence of sleep loss [1,4,32,44]. Thus, we asked whether enriched social experience would alter ppERK levels. As seen in Figure 4A, *Cs* flies exposed to an enriched social environment for 5 days show a significant increase in daytime sleep compared to their isolated siblings, as previously described [2,45]. Interestingly, flies exposed to the enriched social environment for 5 days also show a large increase in ppERK/totERK levels compared to their socially isolated siblings (Figure 4B). Quantification using western blot data confirmed that social enrichment resulted in a statistically significant increase in ppERK levels (Figure 4C). Importantly, *rl* ^1^ mutants do not respond to social enrichment with an increase in sleep (Figure 4A Inset). To determine whether activated ERK alone may be sufficient to increase structural plasticity, we quantified the number of PDF-positive terminals in RU+ treated *+*;*UAS-ERK* ^*SEM*^ */+* ;*GSw-elav/+* flies compared to their RU- fed siblings. As seen in Figure 3D-F, ERK activation significantly increased the number of PDF-terminals in projections from the large ventral lateral neurons. 

**Figure 5:**
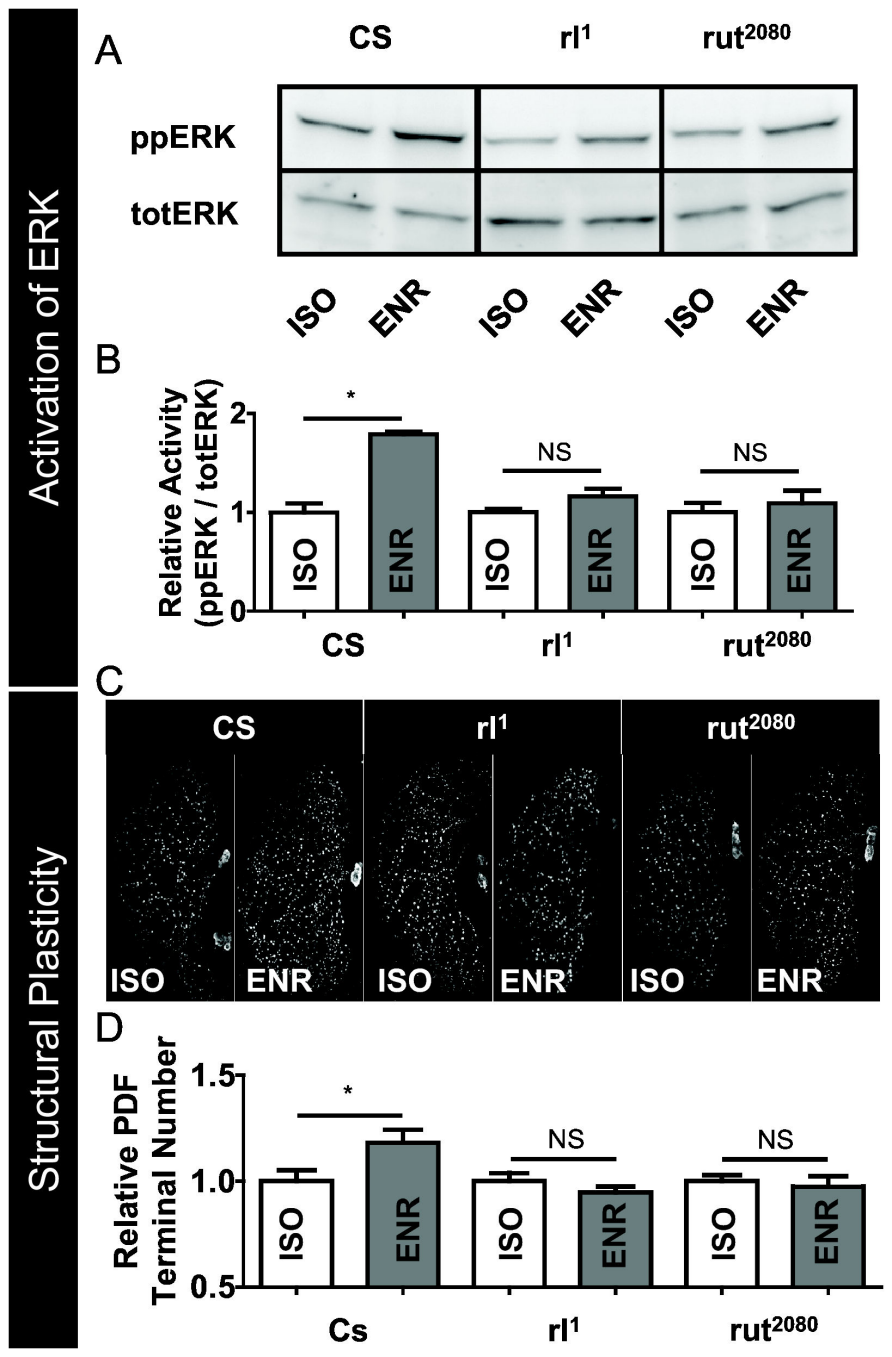
*rl* ^1^ and *rut* ^2080^ have deficits in ERK activation and structural plasticity in response to social enrichment. *A*, In contrast to *Cs* controls, flies mutant for *rl* ^1^ or *rut* ^2080^ do not exhibit an increase in ERK activation following social enrichment. Representative Western blot for ppERK and totERK in flies exposed to social enrichment and social isolation (4 heads/lane). *B*, Quantification of the Western blot in (*A*) for ppERK/totERK levels reveals a statistically significant increase in ppERK/totERK levels in the socially enriched *Cs* flies (p = 0.0002) with no statistical increase in ppERK/totERK levels in either *rl* ^1^ or *rut* ^2080^ mutant flies (p = 0.113 and p = 0.579, respectively, student’s t-test, * = p < 0.05). *C*, Immunohistochemistry for PDF in brains of *Cs*, *rl* ^1^ and *rut* ^2080^ flies exposed to social enrichment and social isolation. *D*, Quantification of PDF-positive terminals from brains in ***C***. PDF terminals are increased following exposure to social enrichment in *Cs* flies p = 0.043, but not in *rl* ^1^ and *rut* ^2080^ flies, *p* = 0.259 and p = 0.666, respectively, student’s t-test; n=11-15/group Values normalized to the isolated siblings.

If the elevations in ppERK levels play a role in mediating the increased structural plasticity, then mutations that attenuate ERK activation should prevent social enrichment from elevating PDF-positive terminals. Since *rl* ^1^ is hypomorphic allele, we first asked whether social enrichment could alter ppERK levels in *rl* ^1^ mutants. As seen in Figure 5A-B, *rl* ^1^ flies do not show an increase in ppERK following social enrichment and do not respond to 5 days of social enrichment with an increase in PDF-positive terminals compared to isolated siblings (Figure 5C-D). To further evaluate the role of ERK in plasticity, we examined the classic learning and memory mutant *rutabaga* (*rut* ^*2080*^), a Ca^2+^/calmodulin-responsive adenylyl cyclase, for its ability to respond to social enrichment with an increase in ERK activation and changes in structural plasticity. Not surprisingly, social enrichment did not alter either ppERK levels or PDF-positive terminal numbers in *rut* ^2080^ mutants compared to isolated controls (Figure 5A-D). These data suggest that increases in ERK activation may play a critical role in mediating changes in structural plasticity following waking experience. 

### Nuclear localization of ERK regulates sleep and cAMP response element (CRE)-mediated transcription 

**Figure 6:**
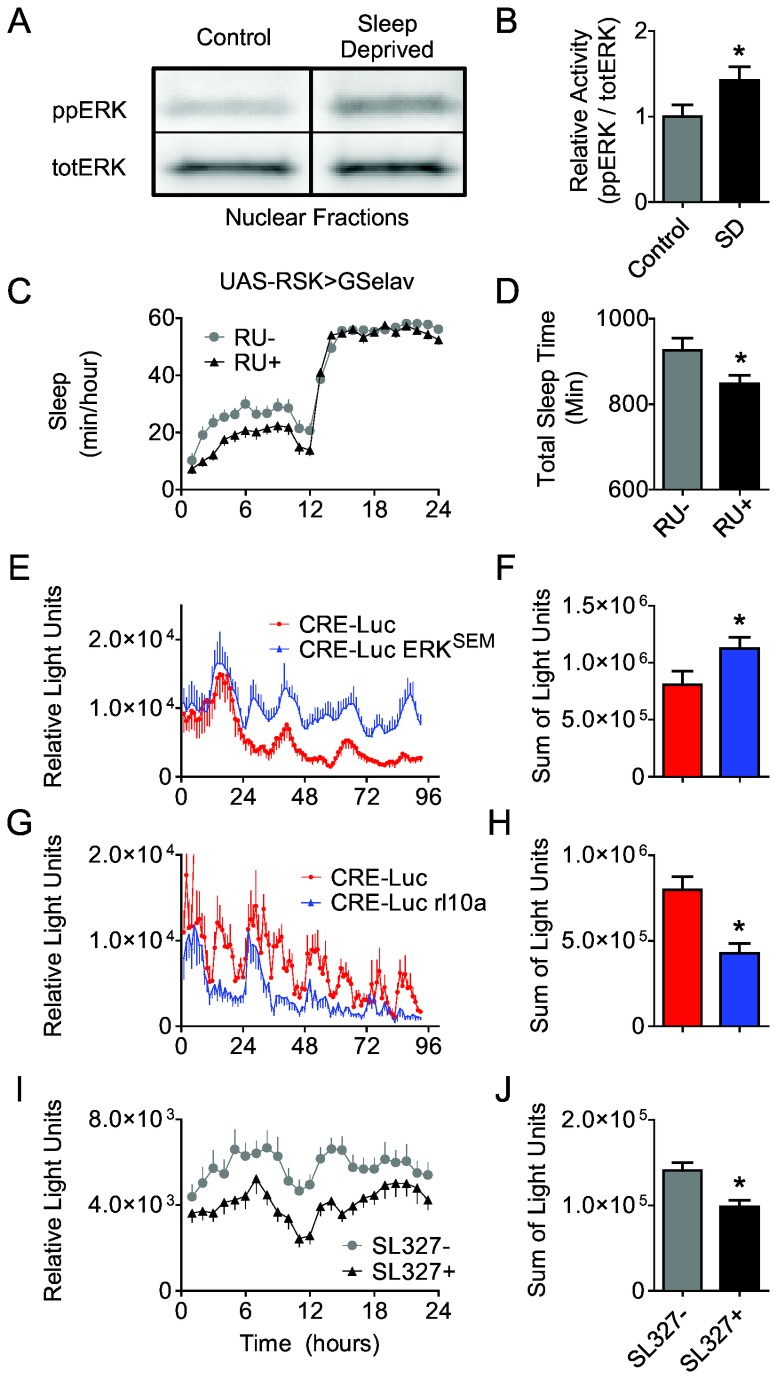
ERK affects CRE-luciferase reporter activity. *A*, Western blot showing an increase in the nuclear localization of ppERK activation following 12 h of sleep deprivation in 5 day old *Cs* flies (40 heads/lane). *B*, Quantification of the Western blot in ***A*** revealed a statistically significant increase in ppERK/TotERK levels, students t-test, p = 0.032, n=8. Data are presented as mean ± SEM. *C*, Sleep profile of flies expressing a wild type version of *UAS-RSK* ^*wt*^ displayed a decrease in sleep. *D*, Total sleep is significantly decreased in RSK over expressing flies. p = 0.0261; student’s t-test, n= 31-32/group. *E*, *elav/Y; CRE-Luc/UAS-ERK* ^*SEM*^ flies (blue trace) have elevated total reporter activity compared to *elav/Y; CRE-Luc/+* controls (red trace); n=19-21/group. *F*, Sum of luciferase activity shown in ***E***; students t-test, p =0.04). *G*, Flies that are heterozygous for the MAPK gene *rolled* (*CRE-Luc/Y; rl* ^10A^ */+*; blue trace) show significant reductions in reporter activity compared to controls (*CRE-Luc/Y; +/CyO*; red trace); n=16-18/group. *H*, Sum of luciferase activity shown in ***G***; students t-test, p = 0.0006). *I*, SL327-fed *elav/Y; CRE-Luc/+* (black trace) show significant reduction in reporter activity compared to vehicle controls (red trace); n=48/group. *J*, Sum of luciferase activity shown in ***I***; students t-test, p = 0.008).

ERK has been shown to play a role in synaptic plasticity by regulating protein synthesis at the level of translation initiation and/or by activating gene transcription [46-48]. In order to activate transcription, activated ERK translocates to the nucleus where it activates downstream transcription factors. To test if sleep deprivation increased levels of activated ERK in the nucleus, we performed western blots on nuclear fractions of fly heads exposed to sleep deprivation for 12 hours. We found that 12 hours of sleep deprivation resulted in an increase in ppERK activation in the nucleus (Figure 6A-B). To further test the hypothesis that ERK activation in the nucleus is required for the behavioral phenotype we conducted experiments to block ERK translocation to the nucleus. When *p90* ribosomal S6 kinase (RSK) is co-expressed with *UAS-ERK* ^*SEM*^, ERK is retained in the cytoplasm preventing its nuclear translocation and the activation of downstream targets [24]. Thus, to determine whether the effects of ppERK are mediated though downstream nuclear events, we expressed *UAS-RSK* ^*wt*^ pan-neuronally in adult flies using *Gsw-elav GAL4*. As seen in Figure 6C-D, RU+ treated ;;*Gsw-elav GAL4/UAS-RSK* ^*wt*^ flies showed a significant reduction in sleep similar to that observed in SL327 fed flies (Figure 1G). These data suggest that activated ERK may be exerting its effects on sleep and plasticity, in part, by nuclear localization and activating gene transcription. To further explore this possibility we examined the ability of *UAS-ERK* ^*SEM*^ to activate cAMP response Element (CRE)-binding protein (CREB) -mediated transcription using transgenic flies carrying a CRE-Luc reporter. As seen in Figure 6E-F, *elav/Y; CRE-Luc/ UAS-ERK* ^*SEM*^ flies displayed a significant increase in bioluminescence compared to genetic controls indicating that ERK can activate downstream gene transcription. Heterozygous loss-of-function flies mutant for ERK show a reduction in CRE bioluminescence (Figure 6G-H). Together these data suggest that nuclear localization of ppERK is sufficient to alter gene transcription. Further, we show that pharmacological inhibition of ERK activation using the drug SL327 in *elav/Y; CRE-Luc/+* flies is sufficient to inhibit CRE bioluminescence (Figure 6I-J). Together these data suggest that ERK influences plasticity, at least in part, by activating transcription.

## Discussion

ERK plays a key role in regulating not only cell differentiation and proliferation during development, but is also critical for regulating long-term potentiation and plasticity related events in the fully developed adult [6-11]. Recent studies have highlighted the important relationship between plasticity induced by waking-experience and sleep need [1,2,4,49,50]. With that in mind, we hypothesized that ERK may provide a molecular link between plasticity and sleep. Since, ERK phosphorylation has been previously correlated with sleep time following *rhomboid* mediated activation of EGFR [19], we over-expressed an active version of ERK pan-neuronally in the adult fly and found a significant increase in sleep. ERK activation increased sleep during the day, was rapidly reversible, and was associated with increased activity during waking. In contrast, disrupting ERK signaling by feeding adults the MEK inhibitor SL327 decreased daytime sleep and lowered waking activity. Together these data indicate ERK activation plays a role in sleep regulation. 

As mentioned, inducing EGFR signaling resulted in an increase in sleep which seemed to correlate nicely with the increase in ppERK[19]. Although these data strongly suggested that the increase in sleep was due to ppERK activation, ppERK was not directly manipulated. Thus our studies confirm and extend the data of Foltenyi and colleagues by demonstrating that directly activating ppERK can increase sleep. While the largest effects of sleep were obtained using a pan neuronal activation of EGFR signaling, Foltenyi also reported that they could map the EGFR induced increase in sleep to the PI. Surprisingly, we did not see any changes in sleep when ppERK was expressed in the PI using the same GAL4 drivers. These data suggest that in the PI, EGFR activation may recruit additional factors along with ppERK to alter sleep. Such regulation may be particularly important for allowing ppERK to carry out multiple functions in various circuits as needed.

In flies, sleep homeostasis is primarily observed during the subjective day [39,40]. Similarly, social enrichment also produces increases in daytime sleep [2,4]. Thus, our data indicate that modulating ERK activity in the adult produces a change in sleep during the portion of the circadian day during which sleep deprivation and social enrichment modify sleep time. Interestingly, ERK activation not only increases daytime sleep, but it also results in the proliferation of terminals in the wake-promoting LNvs [34,51]. The ability of ERK activation to increase synaptic terminals is reminiscent of the change in synaptic markers and structural morphology that are independently observed following sleep deprivation and social enrichment [1-4]. Interestingly, *arouser* mutants show both enhanced ethanol sensitivity and an increase in terminals from the LNvs through its activation by EGFR/ERK [52]. Given that a well characterized function of ERK is to regulate synaptic morphology [53], our results suggest that ERK activation may be a common mechanism linking waking experience, plasticity and sleep.

Although sleep deprivation produces dramatic increases in ERK activation, it is possible that the changes in ERK might be due to stress or non-specific effects of the deprivation apparatus. Such a possibility is unlikely for several reasons. First, changes in ERK activation are not observed in flies that are exposed to mechanical perturbations that do not disrupt sleep [23,40,41]. Secondly, ERK activation is not modified in flies that are subjected to oxidative stress. Finally, ERK activation is not altered during waking induced by starvation. This latter result is relevant given that, in contrast to sleep deprivation, starvation produces prolonged episodes of waking that are not accompanied by a sleep rebound or learning impairments [42]. Thus, ERK activation does not appear to be associated with stress but is tightly correlated with manipulations that alter sleep time. Indeed, RU+ fed *+*;*UAS-ERK* ^*SEM*^ */+* ;*GSw- elav/+* flies responded to sleep deprivation with significantly higher sleep rebound further emphasizing the role of ERK in regulating sleep. A caveat is that the MEK inhibitor, SL327, did not reduce the sleep rebound as predicted. Given the large increases in ppERK levels that are seen throughout the brain in sleep deprived flies, it is possible that SL327 was simply not able to fully disrupt ERK activation, or there is a neuronal circuit-specific role for ppERK which differentiates its effects on baseline sleep from the homeostat. Thus, to further evaluate the role of ERK, we sleep deprived *rl* ^1^ mutants and evaluated both sleep rebound and levels of ppERK/totERK. Interestingly, sleep deprivation did not result in either a sleep rebound or changes in ERK activation in hypomorphic *rl* ^1^ mutants. It is interesting to note that, in contrast to wild-type flies, *rl* ^1^mutants do not respond to either sleep deprivation or social enrichment with an increase in terminals from the LNvs. Thus, while we cannot rule out the possibility that the lack of a sleep rebound might be due to changing *rl* during development, our data are consistent with a role for ERK in sleep regulation in the adult brain. 

As mentioned, ERK activation has been correlated with sleep time following *rhomboid* mediated activation of EGFR [19]. Interestingly, in that study, ppERK was not detectable in cell bodies following *rho* mediated increases in sleep suggesting that, during *rho* activation, ppERK might be modifying sleep at the level of translation initiation [19]. Our data extend these observations and provide genetic evidence that ERK activation may also play a role in regulating sleep and plasticity by activating gene transcription. That is, pan-neuronal expression of RSK, which retains ERK in the cytoplasm and prevents its nuclear translocation [24], results in a decrease in daytime sleep similar to that observed in wild type flies fed SL327. Although previous studies have established a link between CREB and sleep [54,55] we evaluated CRE-Luc solely as a reporter of transcriptional activation. Our data indicates that the expression of *UAS-ERK* ^*SEM*^ increased CRE-Luc activity. In contrast, transgenic CRE-Luc reporter flies show reduced bioluminescence when crossed into a *rl* ^10a^ mutant background. Finally, flies fed SL327 also showed a reduction of CRE-Luc activity. These data are consistent with a recent report demonstrating that activating MEK increases bioluminescence in flies carrying a CRE-Luc reporter [56]. Together these data suggest that ERK activation may alter plasticity and sleep, in part, by activating gene transcription. 

Interestingly, expressing *UAS-ERK* ^*SEM*^ in PDF neurons does not change the number of PDF-terminals and does not alter sleep time. This is in contrast to the effects of expressing *UAS-ERK* ^*SEM*^ pan-neuronally which increases both the number of PDF-terminals and increases sleep. These data suggest that ERK activation can either influence PDF neurons in a non-cell autonomous fashion or that ERK activation is required in multiple circuits to modulate plasticity. Indeed, we have recently shown that increasing sleep by activating the dorsal Fan Shaped Body significantly reduces the number of PDF-terminals [32]. Thus, PDF terminal number provides an accessible read-out of brain plasticity that can be used to elucidate molecular mechanisms linking sleep and plasticity at the circuit level.

It is important to note that in flies there is a critical window of adult development that can influence sleep and learning [2,4,57]. For example, 0-3 day old *rut* ^2080^ mutants are able to respond to social enrichment with an increase in sleep but their older siblings (>3days) cannot [45]. In other words, *rut* ^2080^ mutants can exhibit higher or lower amounts of sleep as adults depending upon environmental context, not levels of *rutabaga per se*. Indeed, *rutabaga* mutants have been reported to have significant variations in sleep (both longer and shorter) compared to controls [55,58]. Given that the environment can stably modify sleep during adult development, even in the absence of memory related genes, care must be taken when classifying a mutant as either long or short sleepers. We should emphasize that we have designed our experiments to avoid making manipulations during this critical time window to avoid such confounds. However, it remains possible that ERK may modify sleep by activating additional downstream targets and/or by regulating translation initiation at the synapse. 

Recent studies have shown that waking experience, including both prolonged wakefulness and exposure to enriched environments, independently produce dramatic increases in both synaptic markers and structural morphology throughout the fly brain and that these changes are reversed during sleep [1-4,45]. To date, most studies have evaluated mutations that disrupt synaptic plasticity to identify the molecular mechanisms linking sleep with plasticity [2,4]. Given that ERK is a key molecule for the regulation of synaptic plasticity and long-term potentiation, we evaluated its ability to alter both sleep and structural plasticity. Our data indicate that both sleep deprivation and social enrichment independently increase ERK phosphorylation in wild-type flies. We also report that expressing an active version of ERK (*UAS-ERK* ^*SEM*^) in the adult fly results in an increase in sleep and an increase in structural plasticity in the LNvs. These data suggest that ERK phosphorylation is an important mediator in transducing waking experience into sleep.

## Supporting Information

Figure S1 A. RU-fed Gsw-elav>UAS-ERKSEM flies show a significant increase in sleep when maintained in Dark:Dark (n=28/group; t-test, p< 0.0001).
*B*. Cs flies maintained in Dark:Dark show significant increases in ppERK following sleep deprivation (n=28/group; t-test, p< 0.0001). C. Representive Western blot of data in *B*.
(EPS)

Figure S2 **Total sleep is not altered in RU-fed *Gsw-elav/+* or *UAS-ERK*^*SEM*^*/+* flies compared to vehicle controls (RU-** **)**
(n = 28-30/group, t-test, p= 0.23 and p= 0.28, respectively.
(EPS)

Figure S3 **RU-fed (RU+) and control (RU-** **) *+*;*UAS-ERKSEM/+* ;*GSw-elav/+* flies show similar arousal thresholds during sleep as indicated by the % flies responding to a single mechanical perturbation delivered during quiescent episodes.**
A 2(RU-, RU+) X ^2^(wake, sleep) ANOVA only revealed a main effect for behavioral state (Wake, Sleep) F[1,24]= 31.27; p= 7.68E-006, but not for treatment (RU-,RU+), F[1,24] =0.042; p= 0.840, and no treatment by behavioral state interaction F[1,24] =0.123; p= 0.729. *<0.05, modified Bonferroni test.
(EPS)

Figure S4 *A*. Total sleep time shown for flies exposed to SL327 at 3 different concentrations. SL327 reduced sleep significantly only at the .250M concentration. *B*. Day bout was also only significantly reduced at the 0.250M concentration. * = p<0.05.
(EPS)
